# Comparison of clinical results of anteromedial and transtibial femoral tunnel drilling in ACL reconstruction

**DOI:** 10.1186/s12891-020-03351-w

**Published:** 2020-06-03

**Authors:** Leena Metso, Kirsi-Maaria Nyrhinen, Ville Bister, Jerker Sandelin, Arsi Harilainen

**Affiliations:** 1 Health Care Center of the City of Helsinki, Työpajankatu 14 A, 00580 Helsinki, Finland; 2 Department of Orthopaedics, Töölö Hospital, Helsinki University Hospital, Helsinki University, Topeliuksenkatu 5, PL 266, 00029 HUS Helsinki, Finland; 3 Department of Orthopaedics, Hyvinkää Hospital, Sairaalankatu 1, PL 585, 05850 Hyvinkää, Finland; 4 ORTON Orthopaedic Hospital, Tenholantie 10, 00280 Helsinki, Finland

**Keywords:** Anterior cruciate ligament reconstruction, Clinical outcome, Anteromedial, Transtibial

## Abstract

**Background:**

A femoral bone tunnel in ACL reconstruction can be constructed from the outside in or from the inside out. When doing it inside out, the approach can be via the anteromedial (AM) portal or through the tibial bone tunnel. It has been suggested that better results might be expected by doing it anteromedially. Clinical results after femoral tunnel drilling via the AM or transtibial (TT) techniques in reconstruction of anterior cruciate ligament (ACL) are presented.

**Methods:**

Three hundred patients with ACL injuries were chosen for this study from previously collected data on ACL reconstructions. They were divided into two groups: 150 patients treated with AM drilling and 150 treated with TT drilling. In the AM group, the reconstructions were performed using a semitendinosus graft with the Tape Locking Screw (TLS™) technique (*n* = 87) or Retrobutton™ femoral and BioScrew™ tibial fixation with a semitendinosus-gracilis graft (*n* = 63). In the TT group, the fixation method used was Rigidfix™ femoral and Intrafix tibial fixation with a semitendinosus-gracilis graft. The evaluation methods were clinical examination, knee scores (Lysholm, Tegner and IKDC) and instrumented laxity measurements (KT-2000™). Our aim was to evaluate if there was better rotational stability and therefore better clinical results when using AM drilling compared to TT drilling.

**Results:**

After excluding revision ACL reconstructions, there were 132 patients in the AM group and 133 in the TT group for evaluation. At the 2-year follow-up, there were 60 patients in the AM group (45.5%) and 58 in the TT group (43.6%). There were no statistically significant differences between the groups in any of the evaluation methods used.

**Conclusion:**

Both drilling techniques resulted in improved patient performance and satisfaction. We found no data supporting the hypothesis that the AM drilling technique provides better rotational stability to the knee.

**Trial registration:**

ISRCTN registry with study ID ISRCTN16407730. Retrospectively registered Jan 9th 2020.

## Background

Arthroscopic-assisted ACL reconstruction has become a standard procedure for controlling anterior-posterior and rotational stability after ACL injuries. In 2018 in Finland, there were 3167 ACL reconstructions reported to the HILMO (Care Register for Health Care) maintained by the Finnish Institute for Health and Welfare [1]. The registry does not differentiate between reconstruction methods, but the vast majority were done using hamstring grafts.

Different fixation methods and approaches have been developed to create bony tunnels. For the femoral tunnel, there are two commonly used drilling techniques. One is the transtibial (TT) drilling technique, in which the tibial tunnel is drilled first. A drill guide is used and positioned intra-articularly at the tibial ACL footprint, and the second quadrant anterior to posterior is targeted. The femoral tunnel is drilled through the tibial tunnel aimed at the posterior fourth quadrant of the femoral condyle in the sagittal plane and at the 10:30 o’clock position in the right knee and the 1:30 o’clock position in the left knee in the frontal plane. Anteromedial (AM) drilling is done from a low anteromedial portal and has been said to provide better rotational stability to the knee by creating a more oblique femoral tunnel positioning [2]. This drilling technique is thought to better mimic the anatomical femoral insertion of the anterior cruciate ligament.

A survey done in 2010 among the members of the American Orthopaedic Society for Sports Medicine disclosed that 70–85% of surgeons use the TT technique of drilling the femoral tunnel through the tibial tunnel [3]. Moreover, Griffin et al. and Fu et al. have presented good and excellent results in 80–95% of cases using the TT technique [4, 5]. Regardless of these good results, the technique has been the subject of criticism. The question is, can the TT technique target the original ACL femoral insertion? Additionally, a nonanatomically positioned graft worsens the function of the knee [6, 7].

The purpose of our study was to determine if there are differences in clinical results after ACL reconstructions performed by either the AM or the TT drilling technique. Our hypothesis was that the AM drilling technique would give better rotational stability and clinical results because the graft placement in the femoral side is claimed to be more anatomical.

## Methods

### Patients

Three hundred patients with anterior cruciate ligament injuries were treated with an arthroscopic reconstruction of the ACL. The patients for this study were chosen retrospectively: 300 consecutive ACL reconstruction patients from our database at the Orton Orthopaedic Hospital, Helsinki, Finland. Sixty of the patients had been included in a previous RCT for comparing fixation methods [8]. The reconstructions took place from January 2006 to August 2011. During that time period, the practice of the clinic changed; therefore, at first the TT drilling technique was used, and afterwards the AM drilling technique was employed. According to the drilling technique, the patients were divided into two groups: 150 ACL reconstructions using the AM drilling technique and 150 reconstructions using the TT drilling technique. Side-to-side laxity measurements were excluded for the patients who had a bilateral ACL tear, whether they were operated on or not. Revision ACL reconstruction was performed on 18 patients in the AM group and on 17 in the TT group. These patients were excluded from the final evaluation, leaving 132 patients in the AM group and 133 patients in the TT group. Ninety patients (68.2%) in the AM group and 86 (64.7%) patients in the TT group attended the 1-year follow-up. At the 2-year follow-up, there were 60 (45.5%) and 58 (43.6%) patients, respectively.

### Surgery

In the AM group, the reconstructions were done using either the TLS™ technique and a semitendinosus graft (*n* = 87) or Retrobutton™ in the femoral tunnel and BioScrew™ in the tibial tunnel with a semitendinosus-gracilis graft (*n* = 63). In the TT group, the fixation methods used were Rigidfix™ in the femoral tunnel and Intrafix™ in the tibial tunnel. The graft used was a semitendinosus-gracilis graft. The results did not reveal any statistically significant differences between the two different fixation methods used in the AM group. All the reconstructions were performed by two experienced knee surgeons (AH, JS).

The TLS™ reconstruction technique has been described by Collette and Cassard [9]. Normally, it is sufficient to use only the semitendinosus tendon as a graft. Bone sockets 10–15 mm deep are made with a hand-powered retrodrill taken through 4.5 mm transtibial and from outside to inside constructed transfemoral drill tunnels. The bone sockets are created doing 360 degree turns while pulling the retrodrill outwards. The graft is pulled inside the knee, the tapes slipped through the tunnels, and the graft is pulled to the right tension. A 10 mm titanium screw is inserted to lock the tapes; the length of the screw is 20 mm in the femur and 25 mm in the tibia.

When using Retrobutton™, the tunnel diameter is the same as the graft’s. A Retrobutton™ loop and implant are attached to the graft, and the graft-implant complex is pulled in to the femoral tunnel. The Retrobutton™ plate is flipped outside the femoral cortex, and the graft is tightened, followed with the tibial fixation by a BioScrew™ of 30 mm length. The screw, the diameter of which is equal to the graft, is inserted eccentrically, compressing the graft against the bony tunnel wall.

The graft in the Rigidfix™ fixation is constructed according to the manufacturer’s instructions: with whipstiches of No. 1 Vicryl (Ethicon Inc., Johnson and Johnson, Somerville, New Jersey) to join the doubled limbs of the semitendinosus and gracilis tendons together. Using drill guides, the depth of the transtibially drilled femoral tunnel is 30–40 mm. With Rigidfix™ instrumentation, two transverse tunnels are drilled for the fixation devices. After the graft is passed in to the femoral tunnel, two Rigidfix™ implants are tapped through the drill guide sleeves transfixing the graft. For the Intrafix™ tibial fixation, No. 1 absorbable whipstitch is used on the graft ends, and the graft is spread across four quadrants between the sleeve and the drill tunnel. After cycling the knee 10 to 15 times, the graft is tightened, and the expansion sleeve and the screw are introduced concentrically to compress the four limbs between the bony tunnel and the device. Three different screw sizes are used: for a graft size up to 8 mm, a 6–8 mm screw is used (7–9 mm screw if the bone quality is suboptimal), and in a graft larger than 8 mm, a 8–10 mm screw is used.

### Post-operative care

Post-operative care was identical for both groups. Immediate mobilization was allowed, and no knee braces were used. Partial weight bearing was allowed immediately and full weight bearing was begun at 2 weeks. Light motion, including on an exercise bicycle, was allowed at 3 weeks. At 6 weeks, active knee extension, deep water running, proprioceptive exercises and weight training was begun with physiotherapists. At 3 months, jumping and jogging were allowed, for which patients were actively trained. From 6 to 12 months after surgery, a gradual return to sports was allowed. After 1 year, there were no restrictions.

### Evaluation methods

To evaluate the results, the subjective International Knee Documentation Committee (IKDC) score (0–100), Lysholm knee score (0–100) and Tegner activity level (0–10) were used. The clinical tests employed were the Lachman and anterior drawer tests to determine anterior laxity and the pivot shift to determine rotatory stability. The Lachman and anterior drawer tests and the pivot shift were graded negative, slightly positive and clearly positive. The anterior-posterior laxity was measured (side-to-side difference with manual maximum force; KT-2000 arthrometer, MEDmetric Corporation, San Diego California) by comparing the injured knee to the control one. These tests and scores were collected before the operation and at the 1- and 2-year follow-ups. Patients also evaluated their activity level before the trauma (Tegner).

### Statistical analysis

The descriptive statistics are presented as means with standard deviation (SD) or counts with percentages. The AM and TT groups were compared preoperatively with a t-test for continuous variables and a Pearson’s chi-squared test for categorical variables. Repeated measures of the changes in outcomes (Tegner, Lysholm, and IKDC scores and side-to side laxity difference evaluations) were compared between the groups with mixed-effects models and an unstructured covariance structure (i.e. the Kenward-Roger method for calculating the degrees of freedom). Fixed effects included the group, the time and group × time interactions. We used age, gender and baseline values as covariates. The repeated measurements were taken at different time points, 1 and 2 years. The mixed models allowed analyses of unbalanced datasets without imputation; therefore, we analysed all available data with the full analysis set. Repeated measures of the pivot shift test were compared between groups with a random-effects logit model, which included age and gender as covariates. A bootstrap method was used when the theoretical distribution of the test statistics was unknown or in the case of a violation of the assumptions (e.g. non-normality). Hommel’s adjustment will be applied to correct levels of significance for multiple testing. Normal distributions were evaluated graphically and with the Shapiro-Wilk W test.

Basic statistical analysis was done using the BMDP Statistical Package (Statistical Solutions Ltd., Cork, Ireland); for more advanced analysis, Stata 16.1 (StataCorp LP; College Station, TX, USA) was used.

## Results

The patients were 12 to 64 years old in the AM group (mean 35 years old) and 13 to 59 years old in the TT group (mean 34 years old). Median time from the injury to surgery was 2 months in the AM group (1 to 42 months) and 3.5 months in the TT group (1 to 366 months). With respect to gender (*p* = 0.2), there was no difference between the two groups.

**Fig. 1:**
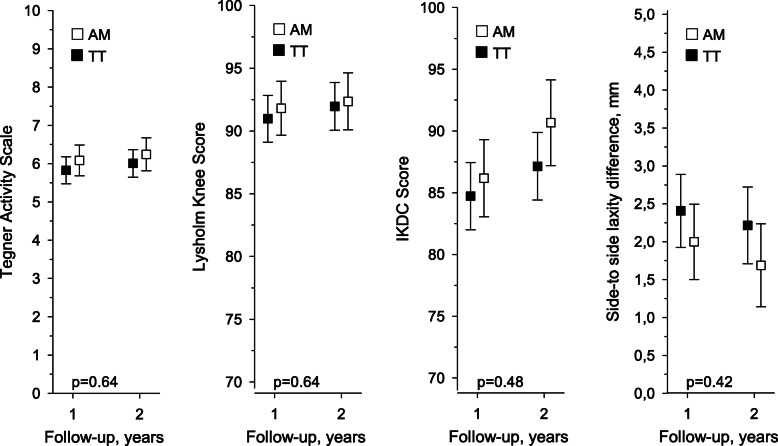
Tegner, Lysholm, and IKDC scores and side-to side laxity difference evaluations 1 and 2 years after ACL reconstruction with AM and TT techniques. The values were adjusted for age, gender and baseline values. The whiskers represent a 95% confidence interval. Hommel’s adjustment will be applied to correct levels of significance for multiple testing

Patients answered questionnaires preoperatively and at 1- and 2-year follow-ups. Improvement was seen on the following tests: Tegner activity levels from preoperative 2 in the AM group and 3 in the TT group to 6 in both groups at the 2-year follow-up, and Lysholm preoperative scores from 73 in the AM group and 76 in the TT group to over 90 points in both groups at the 2-year follow-up. The IKDC scores improved in both groups between the 1- and 2-year follow-ups (Table 1 and Fig. 1). No statistically significant differences between the groups were found. For the KT-2000 laxity measurements, there was a 5.25 mm preoperative difference compared to the non-injured knee in both groups. At the 2-year follow-up, this difference diminished to 1.7–2.2 mm (Table 1 and Fig. 1).

**Table 1:** Tegner, Lysholm, and IKDC scores and side-to side laxity difference evaluations preoperatively before ACL reconstruction with AM and TT techniques, mean + − SD

	AM	TT	P-value
Tegner Activity Scale, mean (SD)	2 (1.4)	3 (1.7)	0.01
Lysholm Knee Score, mean (SD)	73 (16.6)	76 (14.4)	0.1
IKDC Score, mean (SD)	55 (15.9)	59 (14.4)	0.1
Side-to side laxity difference, mm, mean (SD)	5.3 (2.6)	5.2 (3.0)	0.9

In the IKDC classification (A to D), the distribution of classes was equal between the two groups at the 1- and 2-year follow-ups. No statistical differences were found (Table 2)

**Table 2:** IKDC classifications 1 and 2 years postoperatively (chi-square) after ACL reconstruction with the AM and TT techniques

	A	B	C	D	P-value
1-year follow-up					
AM	41	43	3	1	
TT	48	32	4	0	0.3
2-year follow-up					
AM	35	19	5	0	
TT	42	12	3	0	0.2

The Lachman test showed more laxity in the AM group at the 1-year follow-up, but without a statistically significant difference. At the 2-year follow-up, this difference had disappeared (Table 3).

**Table 3:** Clinical stability (Lachman) evaluation 1 and 2 years postoperatively (chi-square) after ACL reconstruction with the AM and TT techniques

Lachman	–	+	++	*P*-value
1-year follow-up				
AM	68	19	2	
TT	76	10	1	0.1
2-year follow-up				
AM	47	8	4	
TT	50	6	1	0.3

The pivot shift test revealed no differences between the two groups, either 1 or 2 years postoperatively (Table 4).

**Table 4:** Pivot shift evaluation 1 and 2 years postoperatively (chi-square) after ACL reconstruction with the AM and TT techniques

Pivot shift	–	+	++	*P*-value
1-year follow-up				
AM	83	5	1	
TT	83	3	1	0.7
2-year follow-up				
AM	51	4	4	
TT	55	1	1	0.1

The follow-up time of our study was 2 years. Originally, we had 132 patients in the AM group and 133 patients in the TT group. Follow-up attendance was poor; at the 1-year follow-up, 68.2% of the patients in the AM group and 64.7% in the TT group visited the hospital. At the 2-year follow-up, attendance was 45.5% in the AM group and 43.6% in the TT group. All our patients were notified of their appointments by letter and given at least two different consultation times. No reasons for not attending were provided.

We compared the baseline characteristics of the follow-up attendees and the drop outs at 1 and 2 years follow-ups.

There were some minor differences in the 1- year follow-up comparison and none in the 2- year follow-up. The attendees of the 1- year follow-up had slightly more laxity in preoperative instrumented laxity measurement. There were more females among those who attended the 1- year follow-up (40%) compared to females among the ones not attending (28%). In the IKDC classification there were 8% in class D of the cases seen 1- year post-operatively, whereas in the ones not attending there were 3.6%. We believe these minor differences do not distort our results. (Table 5).

**Table 5:** Comparison of presurgery measurements between the 1 and 2 year follow-up attendees and drop outs after ACL reconstruction with AM and TT techniques

	Attending 1 year follow-up	Not Attending 1 year follow-up	*P*-value
Age	35	33	0.3
Tegner preinjury	6.9	6.7	0.4
Tegner presurgery	2.9	2.9	0.9
Lysholm presurgery	74	74	0.7
IKDC score presurgery	57	55	0.5
Side-to-Side laxity (mm) manual max.	5.3	4.8	0.03
Sex, male / female	104 / 72	64 / 25	0.04
IKDC classification B / C / D	0 / 153 / 14	2 / 78 / 3	0.05
Lachman test +/++	1 / 175	3 / 86	0.07
Anterior Drawer +/++	3 / 173	4 / 85	0.1
Pivot Shift +/++	2 / 174	3 / 86	0.2
	Attending 2 year follow-up	Not Attending 2 year follow-up	P-value
Age	34	34	0.8
Tegner preinjury	6.9	6.7	0.5
Tegner presurgery	2.9	2.9	0.7
Lysholm presurgery	75	74	0.5
IKDC score presurgery	58	55	0.1
Side-to-Side laxity (mm) manual max.	5.3	4.7	0.1
Sex, male / female	74 / 44	94 / 53	0.8
IKDC classification B / C / D	0 / 100 / 7	2 / 131 / 10	0.4
Lachman test +/++	1 / 115	3 / 146	0.4
Anterior Drawer +/++	2 / 114	5 / 144	0.4
Pivot Shift +/++	1 / 115	4 / 145	0.2

## Discussion

In a study similar to ours that analysed active soccer players, Alentorn-Geli et al. found a statistically significant difference in favour of the AM technique. This was thought to result from a more anatomical insertion of the graft in the femoral side. In the 1- and 2-year follow-ups, there were significantly better results on the Lachman and KT-2000™ arthrometer tests in the AM group, although this difference was lost at the 3- to 5-year and 6- to 10-year follow-ups [10].

Kopf et al. have suggested that a graft placed too superiorly in the femoral lateral wall does not provide the same rotational stability of the knee that a more horizontally placed graft insertion does [11]. Rotational instability and anterior laxity may lead to premature knee arthrosis. Laxity of the ACL causes wear in the posterior and medial parts of the tibial cartilage, and, if the hamstring muscles are weak, they cannot oppose the subluxation occurring from the action of the quadriceps muscle [12]. For the ACL graft to be placed anatomically in the femur, AM drilling is thought to be the best option [13]. Additionally, by moving the tibial tunnel medially and proximally closer to the joint line, it is possible to make the tibial tunnel more horizontal, allowing the femoral tunnel to be drilled closer to the ACL’s anatomical insertion site [14]. Chhabra et al. found similar results in their study [15].

Chang et al. showed in their cadaver study that a horizontally and anteromedially-drilled femoral tunnel is clearly shorter in the lateral femoral condyle than a tunnel drilled transtibially. This must be taken into account when deciding on the femoral fixation method. When using cross-pin femoral fixation, the cross-pin may miss the femoral tunnel, thus weakening the graft’s attachment to the bony tunnel and the tensile strength of the graft [16]. These same conclusions were found in a study by Bedi et al. [17].

Based on cadaver studies, it appears that in ACL reconstructions a more horizontally placed graft provides more stability and more efficiently eliminates rotational instability of the knee [16, 17]. However, the TT technique has been used with good results for many decades [17–20].

Using the Danish Knee Ligament Reconstruction Register, Rahr-Wagner et al. found an increased risk of revision when the AM technique was used for creating the femoral tunnel when compared with the TT technique. According to the authors, one plausible explanation is that the AM technique is a newer and more complex procedure leading to more technical failures and thus a higher revision rate compared with the TT technique [21].

Rotational instability can potentiate the risk of re-rupturing the ACL. Pivot shift is a clinical test for this instability. In our study, pivot shift was slightly positive or positive in 8 cases from the AM group (13.6%) and in 2 cases from the TT group (3.5%) at the 2-year follow-up. There was no statistical difference (*p* = 0,1).

In our previous study, no statistical difference was found between these two drilling methods, although the study’s intent was to compare different fixation methods [8]. Patients with high demand, such as top league soccer players, had better results when using a more anatomic insertion site in the femur (AM drilling), and thus it may be the method of choice for top athletes when uncompromised rotational stability is needed [10]. Our results, nevertheless, suggest that the best clinical results are achieved when operating with the method that is best known and handled properly.

## Conclusions

In contrast to existing studies, there was no evidence of one or the other of the femoral tunnel drilling techniques being better in controlling the rotational instability of the knee after ACL reconstruction. Both drilling techniques resulted in improved patient performance and satisfaction.

## Data Availability

The data supporting the results reported in this article are kept and stored at the ORTON Orthopaedic Hospital, Helsinki, Finland.

## Abbreviations

ACL: Anterior cruciate ligament
TT: Transtibial
AM: Anteromedial
IKDC: International Knee Documentation Committee
TLS: Tape locking screw
SSD: Side-to-side laxity difference
